# Effects of Extraction Methods on the Bioactivities and Nutritional Value of Virginia and Valencia-Type Peanut Oil

**DOI:** 10.3390/molecules27227709

**Published:** 2022-11-09

**Authors:** Zineb Lakhlifi El Idrissi, Hamza El Moudden, Najoua Mghazli, Abdelhakim Bouyahya, Chakir El Guezzane, Mohammed Merae Alshahrani, Ahmed Abdullah Al Awadh, Khang Wen Goh, Long Chiau Ming, Hicham Harhar, Mohamed Tabyaoui

**Affiliations:** 1 Laboratory of Materials, Nanotechnology, and Environment, Department of Chemistry, Faculty of Sciences, Mohammed V University, Rabat BP 1014, Morocco; 2 Higher School of Technology of El Kelaa Des Sraghna, Cadi Ayyad University, El Kelaa Des Sraghna BP 104, Morocco; 3 Microbiology and Molecular Biology Team, Faculty of Sciences, Mohammed V University, Rabat BP 1014, Morocco; 4 Laboratory of Human Pathologies Biology, Department of Biology, Faculty of Sciences, Genomic Center of Human Pathologies, Faculty of Medicine and Pharmacy, Mohammed V University, Rabat BP 1014, Morocco; 5 Department of Clinical Laboratory Sciences, Faculty of Applied Medical Sciences, Najran University, 1988, Najran 61441, Saudi Arabia; 6 Faculty of Data Science and Information Technology, INTI International University, Nilai 71800, Malaysia; 7 Pengiran Anak Puteri Rashidah Sa’adatul Bolkiah Institute of Health Sciences, Universiti Brunei Darussalam, Gadong, Bandar Seri Begawan BE1410, Brunei

**Keywords:** extraction method, peanut oil, phytosterols, radical scavenging capacity, tocopherols, Virginia, Valencia

## Abstract

This study aimed to evaluate the effects of peanut varieties cultivated in Morocco (*Virginia* and *Valencia*) and extraction methods (cold press, CP; Soxhlet, Sox and maceration, and Mac) on the fatty acid profile, phytosterol, and tocopherol contents, quality characteristics, and antioxidant potential of peanut seed oil. The DPPH method was used to determine the antioxidant activity of the oils. The results revealed that fatty acid content was slightly affected by the extraction technique. However, the CP method was shown to be an excellent approach for extracting oil with desirable quality features compared to the Sox and Mac methods. Furthermore, the peanut oil extracted via CP carried a higher amount of bioactive compounds and exhibited remarkable antioxidant activities. The findings also revealed higher oleic acid levels from the *Virginia* oil, ranging from 56.46% to 56.99%. Besides, a higher total phytosterol and tocopherol content and DPPH scavenging capacity were obtained from the *Valencia* oil. Analyzing the study, it can be inferred that extraction method and variety both affect the composition of the peanut oil’s bioactive compounds and antioxidant activity. This information is relevant for extracting peanut oil with a greater level of compounds of industrial interest.

## 1. Introduction

Peanut (*Arachis hypogaea* L.), known also as groundnut, is a legume crop native to South and Central America and is now a major food crop worldwide for both humans and animals, owing to its availability and affordability compared to other types of nuts [1,2]. It is cultivated throughout the tropics and subtropics but is mainly grown in Asia and Africa. Peanuts are the world’s fourth-largest oilseed crop after soybeans, rapeseed, and cotton [3]. From 2020–2021, 49.62 million metric tons (MMT) of peanuts were produced globally, contributing approximately 6.43 MMT of oil to total worldwide vegetable oil production. China (17.9 TMT) and India (6.7 TMT) are the top two peanut producers [3]. In Morocco, about 25.000 hectares of peanuts are grown on the Atlantic coast between Kenitra and Larache [4]. There is a wide diversity of peanut cultivars, such as Manfredi, GK-7, Langley, and Okrun. Nevertheless, the four main varieties (Runner, Virginia, Spanish, and Valencia) have gained market acceptance because of their flavor, oil content, size, form, and disease resistance [5]. Peanut is an abundant source of lipid, protein, dietary fiber, and minerals (essential for human nutrition), including iron, zinc, potassium, and magnesium. This plant is widely used as a dietary ingredient, protein supplement, or to enhance the functional characteristics of foods due to its high nutritional attributes [1]. Peanut oil is used in cooking, manufacturing margarine, shortening, and producing soap [2]. Besides, seeds are primarily consumed directly, either raw or roasted, ground into peanut butter, or inserted into various foods like snacks and confectionery products for nutritional benefits [5]. The current literature reports suggest that peanut consumption is correlated to a reduced risk of coronary heart disease (CHD), gallstones in both sexes, certain types of cancers, diabetes, and improved weight management [1,6,7]. Therefore, its use in human nutrients can be considered as an important functional food.

Peanut oil (PO) comprises 80% unsaturated fatty acids, predominantly monounsaturated fats (MUFA), and 20% saturated fatty acids (SFA) [5]. Clinical investigations documented that the intake of MUFA and PUFA were beneficial to human cardiovascular systems and can effectively lower hyperlipidemia and harmful cholesterol [7]. Like other nuts, its principal fatty acid constituents are oleic acid, linoleic acid, and palmitic acid [1]. Studies have reported that regular U.S. peanuts contain 49% to 57% of MUFA, while medium and high oleic peanuts comprise 66% to 69% and 78% to 80% of MUFA, respectively [8]. Further, a high-oleic peanut has a longer life span than a low-oleic peanut and also greater flavor quality and stability [1].

PO contains a substantial amount of phytosterols (207 mg/100 g), which is greater than olive oil’s phytosterol level [7]. β-sitosterol is the most abundant phytosterol (PS) in peanuts, reaching 61–114 mg/100 g in roasted peanuts [9]. In addition, peanut oil provides a precious source of lipid-soluble vitamins, such as tocopherols (Vitamin E) [6]. These compounds are recognized for acting as antioxidants, maintaining cell membrane stability against oxidative stress [6]. Compared to other nuts, peanuts have the highest level of total tocopherol and are rich in the individual tocopherol [7]. The α-tocopherol concentration of peanuts is 20.21 mg/100 g, which is slightly lower than almonds (25.65 mg/100 g) but significantly greater than other tree nuts [2]. 

Extraction of lipid and other valued substances is strongly reliant on the method employed for extraction [10]. Therefore, an appropriate extraction technique is required to yield the desired components. Pressing, the Soxhlet system, and the combined use of pre-pressing and solvent extraction are the three most prevalent processes for producing oils [11]. Solvent/Soxhlet extraction may affect oil features and induce partial deterioration of most of the minor phytochemicals, which have a wide range of functional, antioxidative, and pro-oxidative effects [12,13]. However, cold-pressing (CP) is preferred as it produces natural edible oils with neither heat nor chemical treatments. Yet, its productivity is low, and it is difficult to obtain a consistent quality product [11].

In the literature, several studies have investigated the use of different methods to extract oil from the peanut seed. These techniques comprise supercritical fluid extraction [14], aqueous enzymatic extraction [15], ultrasonic extraction [16], extraction by cold press (CP), and extraction by Soxhlet (Sox) [11]. However, neither of these reports has explored the comparison of the physicochemical parameters and antioxidant activity of PO as affected by several extraction procedures. 

This work aimed to assess the impact of the extraction method (cold press, CP; Soxhlet, Sox and maceration, and Mac) and the varieties cultivated in Morocco (Virginia and Valencia) on the oil content, fatty acid profile, quality characteristics, phytosterol, and tocopherol contents, and the antioxidant activity of peanut oil. In addition, the data were submitted to principal component analysis (PCA) as means of identifying any discrimination among the samples.

## 2. Materials and Methods

### 2.1. Plant Material

Two locally available peanut varieties (Virginia and Valencia) were purchased from the local ‘Al Weaam’ market in Kenitra, Morocco (34°15ʹ0″ N 6°34ʹ59.999″ W). The Virginia type has the largest seed size, while the Valencia type contains three or more small seeds per pod. The peanut hulls were removed to recover peanut seed kernels. Then, the kernels were hand-selected to eliminate damaged seeds (broken or cracked seeds). 

### 2.2. Chemicals and Reagents

All chemicals and solvents of analytical and HPLC grade utilized in this study were sourced from Professional Lab (Casablanca). Vitamin E homologues (α, β, γ, and δ) and the standard fatty acid methyl ester mixture (FAME) used for chromatographic analyses were supplied by Sigma-Aldrich (St. Louis, MO, USA). All the glassware (Soxhlet, condenser, round bottom flask, funnel, büchner funnel, erlen-meyer flask and büchner flask) was made of borosilicate glass and purchased from Borosil Scientific Glassware, Mumbai, India.

### 2.3. Extraction Procedure

#### 2.3.1. Cold Press Extraction

Cold pressing of the peanut oil was performed with a screw expeller (IBG Monforts Oekotec GmbH, Mönchengladbach, Germany) at a temperature of 90 °C, according to El Bernoussi et al. [17]. After extraction, oil samples were centrifuged (Anke KA-1000, Shanghai, China) at 4000 rpm for 10 min to eliminate impurities and transferred into amber bottles. Then, the crude oil was stored at 4 °C and preserved from sunlight until analysis.

#### 2.3.2. Soxhlet Extraction

Samples of peanut kernels were milled to a fine powder using an electric blender (Moulinex, model LM422125, Lyon, France) and then passed through a 60-mesh seize. In reference to Nasri et al. [18], 50 g of peanut powder was transferred into a cellulose paper cone and extracted with n-hexane (350 mL) in a Soxhlet apparatus for 8 h at 60 °C. The lipid fraction was collected in the hot solvent as the extraction solvent is cycled continuously through the matrix by boiling and condensation. After the extraction was accomplished, the solvent was eliminated with a rotary evaporator (model VV2000, Heidolph, Schwabach, Germany) under a vacuum at 50 °C. The obtained oil was dehydrated by passing through a funnel containing Whatman No. 2 filter paper and anhydrous sodium sulfate.

#### 2.3.3. Maceration Extraction

A slightly modified cold maceration method described by Eddahhaoui et al. was used [19]. Briefly, 50 g of peanut powder was combined with 400 mL of n-hexane and extracted by maceration using a magnetic stirrer (VELP AREX CerAlTop™, Usmate Velate, MB, Italy) for 48 h. The mixture was filtered through Whatman 85 No. 1 paper filter with a Büchner funnel under vacuum. A rotary evaporator (model VV2000, Heidolph, Schwabach, Germany) was used to remove the solvent under reduced pressure at 50 °C. The extracted oils were subsequently weighed and placed in brown glass bottles, then conserved in a refrigerator at 4 °C until further analysis.

### 2.4. Analytical Methods

#### 2.4.1. Quality Characteristics

The acid value (AV), peroxide value (PV), extinction coefficients (E_232_ and E_270_), and p-anisidine value (p-AnV) were evaluated according to the International Organization for Standardization (ISO) analytical methods [20,21,22,23], respectively.

AV was measured by titration of an oil solution in a 1:1 (*v*/*v*) ethanol/ether mixture with an ethanolic solution of KOH and represented as milligrams of KOH per gram of oil (mg KOH/g oil). PV was measured by iodine titration of an oil solution in a 2:1 (*v*/*v*) iso-octane/acetic acid mixture with a sodium thiosulfate solution and represented as milliequivalents of active oxygen per kilogram of oil (meq O_2_/kg oil). E_232_ and E_270_ were determined in a 10 mm cuvette, utilizing an LLG-uniSPEC 2 spectrometer (LLG Labware, Meckenheim, Germany) of a 1% (*w*/*v*) oil solution in cyclohexane. p-AnV was measured by spectrophotometry at 350 nm of a solution comprising 0.5 g of oil dissolved in 25 mL of iso-octane, and the total oxidation or TOTOX value was calculated according to Equation (1):

TOTOX = 2PV + p-AnV(1)

#### 2.4.2. Pigment Quantification

The carotenoid and chlorophyll contents of the oils were assessed using the procedure outlined by Espínola et al. [24]. In brief, 7.5 g of POs were dissolved in 25 mL of cyclohexane and the absorbance values of chlorophyll and carotenoid were measured at 670 and 470 nm, respectively, using an LLG-uniSPEC 2 spectrophotometer (LLG Labware, Meckenheim, Germany). The results were computed according to Equations (2) and (3):

(2) $$\mathrm{Chlorophyll}\;(\mathrm{mg}/\mathrm{kg})=\frac{\left(A_{670\;}\times 10^{6}\right)}{\left(613\times 100\times d\right)}$$

(3) $$\mathrm{Carotenoid}\;(\mathrm{mg}/\mathrm{kg})=\frac{\left(A_{470\;}\times 10^{6}\right)}{\left(2000\times 100\times d\right)}$$

The chlorophyll concentration is represented as mg of α-pheophytin per kilogram of peanut oil, and the carotenoid concentration is displayed as mg of Lutein per kilogram of peanut oil.

#### 2.4.3. Fatty Acid Profile

The fatty acids of the PO samples were trans-esterified with a methanolic solution of potassium hydroxide to their corresponding methyl esters, following the ISO 12966-2 method [25]. Determination of peanut fatty acids profile was carried out using gas chromatography, furnished with an FID and a capillary column. The carrier gas utilized was helium at a flow rate of 1.0 mL/min. A sample split ratio of 1:50 was used, and the injection volume was 2 µL. The oven temperature was initially set at 165 °C for 8 min and scheduled to rise to 210 °C at 2 °C/min. Fatty acids were identified on the basis of retention time as compared with those of authentic standards, and the findings were reported as a percentage of total fatty acids.

#### 2.4.4. Phytosterol Composition

The phytosterols were quantified following the ISO 12228–1 method utilizing a Varian 3800 gas chromatograph, furnished with a VF-1 ms column (30 m; 0.25 mm i.d.) [26]. Helium was used as the porter gas with a total gas flow rate of 1.6 mL/min. The column was maintained at a constant temperature of 270 °C. The injector and detector temperature were regulated at 300 °C. In a splitless mode, the sample injection volume was 1 µL. A Varian Star Workstation v 6.30 was used in order to process the data (Varian Inc., Walnut Creek, CA, USA).

#### 2.4.5. Tocopherols Content

Tocopherol analysis was performed as previously described by El Moudden et al. [27]. An amount of 0.25 g of peanut oil was dissolved in 25 mL of n-hexane and combined using a vortex mixer. The samples were passed through a 0.45 μm PTFE membrane and inserted into vials for analysis. The detection was conducted using an HPLC system (Waters e2695, Milford, MA, USA), including a fluorescence detector with excitation and emission wavelengths fixed at 290 nm and 330 nm, respectively. The separation was carried out on a C18-Varian column (25 cm × 4 mm; Varian Inc., Middelburg, Netherlands). The mobile phase was isopropanol/isooctane (1:99, *v*/*v*) at a flow rate of 1.2 mL/min. Tocopherols were identified and quantified using standard solutions and calibration curves. The data were represented as milligrams of tocopherol per kilogram of oil (mg/kg oil).

#### 2.4.6. Radical Scavenging Activity (RSA)

The ability of POs to scavenge DPPH free radicals was evaluated using the procedure previously described by Suri et al. [28]. In brief, 1 mL of 10% (*w*/*v*) oil solution in 3 mL ethyl acetate was vortexed for 30 s with 4 mL of DPPH solution (0.1 mM) in ethyl acetate. After 30 min of incubation, the absorbance of the control solution of DPPH and oil reaction mixtures was measured at 515 nm with an LLG-uniSPEC 2 spectrophotometer. The RSA toward DPPH was computed using Equation (4):

(4) $$\mathrm{RSA}\;(\%\;\mathrm{inhibition}\;\mathrm{DPPH})=(1-\frac{A_{30}}{A_{C}})\times 100$$

where A_C_ and A_30_ represent the absorbance of the control DPPH solution and sample solutions, respectively.

### 2.5. Statistical Analysis

All analytical experiments were carried out for three replicates, and the data were expressed as the average of the three measurements with the standard deviation (mean ± SD, n = 3). The findings were submitted to analysis of variance (ANOVA) and Tukey’s test performed with IBM SPSS Statistics Version 21 software (SPSS Inc., Chicago, IL, USA). Statistical significance was defined as a *p*-value < 0.05. Also, Pearson’s test was employed to perform correlation analysis. The content of the phytochemical compounds (pigment contents, PUFA, total sterols, and total tocopherols) and analytical parameters (AV, PV, p-AnV, TOTOX, E_270_, and E_232_) was analyzed using principal components analysis (PCA) in order to determine any discrimination amongst the samples in terms of extraction method and peanut varieties. In this analysis, PCA was accomplished by utilizing XLSTAT 2014 software (Microsoft^®^, Redmond, WA, USA).

## 3. Results and Discussions

### 3.1. Quality Characteristics

The AV analysis reveals the storage circumstances of the oil, given the fact that the oxidizing elements, including heat, sunlight, oxygen, and metals, can enhance the degradation of triacylglycerols, inducing rancidity and the emergence of free fatty acids [29]. Table 1 illustrates the quality characteristics of the PO samples extracted by different methods.

In the current investigation, the PO extracted using the Mac method exhibited higher acid values (0.81 and 0.80 mg KOH/g for VIO and VAO, respectively) than that of the Sox and CP methods. This finding might be attributed to the peanut seeds being subjected to high temperatures and extended extraction durations. Similarly, earlier studies have reported a higher AV obtained from solvent-extracted black cumin and red pepper seed oils [30,31]. All PO samples had an AV of less than 4 mg KOH/g, which is within the range of the adequate level for edible oils [32].

The primary oxidation products were evaluated by examining their PV and specific absorption at 232 nm, as pointed out by Gharby et al. [33]. PV is undeniably a good predictor of oil quality and freshness [34]. Clearly, a high PV in plant oils indicates overheating or improper conservation conditions [35]. The results showed that the PV of the POs generated by different extraction methods ranged from 2.15 to 12.9 meq O_2_/kg and 3.7 to 11.8 meq O_2_/kg for VIO and VAO, respectively. Furthermore, Sox extracted PO displayed the most significant value for PV (12.9 meq O_2_/kg for VIO and 11.8 meq O_2_/kg for VAO), which is higher than the average value established by the Codex Alimentarius Commission, which states a maximum permissible PV cannot exceed 10 meq O_2_/kg for vegetable oils [32]. The observed increase in PV in the present study might be a result of the formation and accumulation of hydro-peroxides due to exposure to high temperatures [35].

Similarly, the E_232_ values adopted a similar variation pattern as PV due to their correlation [33]. As a matter of fact, E_232_ was found to be higher in those POs extracted by the Sox method (1.510 and 1.434 for VIO and VAO, respectively) in comparison with the other two processes (Mac and CP). These outcomes were generally in accordance with those documented by Chouaibi et al. [31] for red pepper seed oil. Regardless, Gharibzahedi et al. [13] stated that the PV of Mac-extracted walnut oil tended to be more significant than the other two techniques (CP and modified Bligh and Dyer MBD). Overall, the primary oxidation products substantially increased with high temperatures as a result of the involvement of hydrolytic enzymes in the hydrolysis of fatty acids [36].

The p-AnV and specific absorbance at 270 nm was used to assess the secondary oxidation products [33]. Undoubtedly, p-AnV permits the quantification of aldehyde compounds, like 2-alkenals and 2, 4-alkenes, resulting from the decomposition of hydroperoxides, which are commonly attributed to the off-flavor of oil [33]. In comparison, the absorbance at 270 nm is used to quantify conjugated trienes and certain secondary products (ketones, α-diketones) [37]. As shown in Table 1, the PO obtained using the Mac method had the greatest p-AnV level (3.78 and 4.69 for VIO and VAO, respectively). In contrast, the lowest p-AnV was related to the CP-extracted PO (0.84 and 0.76 for VIO and VAO, respectively), which were substantially different (*p* ≤ 0.05). Accordingly, with p-AnV, the specific extinction coefficient, E_270_, of those POs generated by the Mac and Sox methods was higher than those obtained by the CP method.

Furthermore, the PV and the p-AnV combination can be used to calculate the TOTOX value [38]. However, TOTOX is an empirical parameter that has the advantage of representing the oxidation state of the fat and its potential to evolve into additional oxidation products [39]. The TOTOX value of high-quality oil is generally less than 10 [40]. Hence, from the data presented in Table 1, the PO generated by the Sox method had a high level of TOTOX (29.01 and 25.74 for VIO and VAO, respectively), while the lowest TOTOX value was associated with the PO obtained via CP, indicating the slow oxidation of the oil. These results revealed that those POs extracted using the Sox and Mac techniques had considerable oxidative status, while those obtained via the CP method had better quality. Gharby et al. [41] outlined a similar trend for black cumin seed oil.

### 3.2. Chlorophyll and Carotenoid Content

Color is an essential indicator of vegetable oil quality [42]. Carotenoids, chlorophylls, and their derivatives are the most common natural pigments present in oils [42]. As seen in Table 1, the levels of carotenoids and chlorophyll assessed in the PO samples extracted using the various procedures showed significant variations (*p* ≤ 0.05).

For each variety, CP-extracted PO presented the highest content of carotenoid (0.51 and 0.29 mg/kg for VIO and VAO, respectively) and chlorophyll (0.72 and 0.42 mg/kg for VIO and VAO, respectively) as compared to the PO obtained by the Sox and Mac methods. These results were lower than those documented by Tuberoso et al. [43] and Falade and Oboh [44] for POs collected from diverse origins. They reported that the carotenoid and chlorophyll content of PO was 1.5 ± 0.1 mg/kg and 4.54 ± 0.02 mg/kg, respectively. The reported results might be attributable to the changes in species and oil manufacturing. However, our findings concur with those obtained by Can-Cauich et al. [45], who documented a higher β-carotene level in pumpkin seed oil when mechanical pressing (MP) was used instead of solvent extraction (OS).

The VIO presented the highest carotenoid and chlorophyll content for each extraction method compared to VAO. According to several researchers, vegetal oil generated by MP without the use of extra and intentional heat provides better preservation of the phytonutrients and bioactive components, including carotenoids, naturally inherent in the oils [46]. Moreover, Bhatnagar-Panwar et al. [47] suggest that carotenoids are located in distinct sections of the peanut kernel’s oil-comprising spherosomes. Thus, the pressed oil’s lighter color relative to the solvent-extracted oil could be attributable to quantitative carotenoids leaching through the oil rather than the solvent [1].

### 3.3. Radical Scavenging Activity (RSA)

Antioxidants in oils promote their oxidative resistance and inhibit their oxidative deterioration by retarding the oxidation process through interaction with free radicals or by limiting the propagation stage by scavenging alkoxy and alkylperoxy radicals [27]. As illustrated in Table 1, significant variations were observed in the inhibiting behavior, expressed as RSA values, of the PO samples obtained from different extraction methods. Indeed, the RSA of CP-extracted PO (47.68% and 50.10% for VIO and VAO, respectively) was more significant than those obtained via the Sox and Mac methods. The finding might be explained by the much lower tocopherol level in the latter. These outcomes were in line with those of Suri et al. [28], who found that the extracted PO using the MP method (60.64%) had a higher DPPH radical scavenging capacity than solvent-extracted oil (58.35%).

For each extraction technique, the antioxidant capacity of the PO was higher for VAO with regard to the values obtained for VIO. This finding can be credited to its high tocopherol concentration, especially γ-tocopherol, which has been previously pointed out to be more stable than its α-homologue, and it is the highest level of phytosterols content, relatively speaking [48]. In agreement with our present finding, some previous studies also revealed that the antioxidant properties of seeds could be ascribed to PUFA, tocopherols, and phenolic compounds [49].

### 3.4. Oil Content and Fatty Acid Profile

The oil yield of both varieties extracted by the Sox method was the highest (56.77% and 47.38% for VIO and VAO, respectively), followed by the Mac (43.38% and 34.81% for VIO and VAO, respectively) and CP extraction methods (36.19% and 30.38% for VIO and VAO, respectively), with considerable variations in oil content among the three extraction methods (Table 2). As a matter of a fact, using thermal treatment disrupts the cohesive and adhesive connections that connect the oil molecules and oil matrix components [43]. These outcomes are consistent with those provided by Suri et al. [28] and Can-Cauich et al. [50], who found a greater yield of peanut and pumpkin oil, respectively, using solvent extraction compared to mechanical extraction. However, Virginia exhibited the highest oil content, regardless of extraction method, compared to Valencia.

The FA composition is an essential marker of the nutritional properties of the oil [33]. As shown in Table 2, the oleic and linoleic acids were found to be the predominant UFAs from all the PO samples. Significant differences (*p* ≤ 0.05) were noticed within primary FAs between the two peanut kernel varieties. The highest rate of oleic acid was found in VIO, varying between 56.46 and 56.99%. VAO recorded the greatest linoleic acid level, ranging from 42.66 to 42.94%. Linolenic acid was present in trace amounts in the POs. In addition, palmitic and stearic acids were the principal SFA, appearing in the proportion of 8.77–9.45% and 3.20–4.38% for VIO and 11.82–12.03% and 3.35–3.59% for VAO, respectively. Our results are consistent with other studies conducted abroad on various peanut species [51,52]. The differences in the FAs profile between peanut cultivars might be attributed to the diversity of the genotypes, kernel maturity, climatic circumstances, growth site, and interactions among these aspects. In addition, research by Nawade et al. [53] revealed that oleic acid increases while linoleic and palmitic acids decrease with seed maturity.

The extraction methods slightly affect the FA composition of the POs. However, a significant change in linoleic acid content was noticed in VIO under differing methods. Our findings were similar to those documented by Al Juhaimi et al. [11] and Suri et al. [28]. Relatedly, other studies by Gharibzahedi et al. [13] and Can-Cauich et al. [50] showed that the FA composition of oils from walnut and pumpkin was not affected by the extraction process.

MUFAs and PUFAs are crucial in the regulation of biological functions as well as the prevention and treatment of a wide range of human diseases, such as heart disease and inflammation [54]. Nevertheless, as the amount of PUFAs increases, an oxidation reaction in the oil is more likely to occur [7]. Our data also highlighted considerable variations in the SFA, MUFA, and PUFA percentages between the two varieties. However, VAO showed the highest long-chain SFA content, comprising 16.55–16.90% of palmitic, stearic, and arachidic fatty acids. A high proportion of MUFA was present in VIO, ranging between 57.54% and 58.18%, which mainly resulted from the highest content of oleic acid. Besides, VAO presented a high level of PUFAs, varying between 42.73% and 43.03%. In fact, the edible oils with a high O/L ratio and low IV were related to longer shelf life and lower rancidity [55]. However, the CP method generated the highest O/L ratio with 2.11% and 0.93 for VIO and VAO, respectively. In this context, the Virginia type of oil was reported to have the greatest O/L ratio, followed closely by the Runner type, implying that these varieties produce better oil quality than Valencia [56].

### 3.5. Phytosterols Composition

Plant sterols, also known as phytosterols, are the main constituents of the unsaponifiable matter of plant oils, representing approximately 60–80% [57]. These compounds are a helpful parameter for detecting adulteration or proof of authenticity [58]. Furthermore, their identification is crucial due to their antioxidant properties and health advantages [59]. Table 3 summarizes the total and individual phytosterol (PS) levels of the PO obtained from the Virginia and Valencia types extracted via three methods. The data are represented in milligrams by oil kilograms (mg/kg). In all evaluated PO samples, the following PSs were detected and quantified: β-sitosterol, campesterol, stigmasterol, cholesterol, Δ-5-avenosterol, Δ-7-stigmasterol, and Δ-7-avenosterol.

The level of total PSs in the POs varied from 1904.16 to 2816.34 mg/kg, which was determined to be within the range of values previously cited in the literature for POs from other nations (900–4344 mg/kg) [1]. Hence, VAO had a higher total sterol content (2272.24–2816.34 mg/kg) in comparison with VIO (1904.16–1970.60 mg/kg). Accordingly, Awad et al. [9] stated that Valencia had the highest amount of PS among the four cultivars (Red, Runner, and Virginia). In another study, Francisco and Resurreccion [60] demonstrated that PS concentration was impacted by variety and maturation, with quantities increasing as maturity progressed. However, a significant difference in the PS content of all the POs tested was observed among the three extraction methods. In contrast to our findings, Gharby et al. [41] stated that the extraction procedure (CP and OS) did not impact the PS content of black cumin seed oil. Moreover, our results revealed that the VAO obtained from the CP method contained the highest amount of PS (2816.34 mg/kg), followed by Mac (2676.88 mg/kg) and Sox (2272.24 mg/kg).

β-sitosterol was shown to be the most prevalent sterol in PO, with contents of 1205.25 to 1277.41 mg/kg and 1473.07 to 1839.42 mg/kg for VIO and VAO, respectively. This PS is also abundantly present in rapeseed, sunflower, and olive oil [27]. Research has shown that β-sitosterol may inhibit colon and breast cancer growth and preserve against heart disease [49]. β-sitosterol contributed the most to total PS levels (63.25–65.34%), followed by campesterol (13.29–15.33%) and stigmasterol (7.21–10.05%) in the POs. The obtained results were slightly lower than those documented by Awad et al. [9] and Jonnala et al. [61] and in line with the data exposed by Carrín and Carelli [1]. In addition, it has been pointed out that these three PSs represent approximately 95% of total peanut sterols [62].

Within each variety, the β-sitosterol (1277.41 and 1839.42 mg/kg for VIO and VAO, respectively) and campesterol (271.65 and 429.71 mg/kg for VIO and VAO, respectively) content of the POs was higher when CP is used instead of the Mac and Sox methods. Further, our results also revealed that, for each technique, the VAO was richer in β-sitosterol and campesterol compared with VIO. Such variations in PS content have already been described in a variety of edible oils after thermal processing [63]. In this context, Oracz et al. [64] noted that the heat treatment causes a decrease in the amounts of free sterol and esterified vegetable oils; this might clarify why the Sox method, in contrast with the CP method, was shown to have a lower PS content. Accordingly, Can-Cauich et al. [45] and Fernández-Cuesta et al. [65] found that employing the MP approach rather than the OS method resulted in a greater level of certain sterols in pumpkin oil and safflower oil, respectively.

The concentration of stigmasterol, one of the major sterols, varied in all of the POs examined and ranged from 178.50 to 190.94 mg/kg and 166.86 to 208.23 mg/kg for VIO and VAO, respectively. Indeed, stigmasterol can exhibit a remarkable pharmacological potential as an antihypercholesterolemic, anti-inflammatory, antioxidant, hypoglycemic, and antitumor agent [66]. Our findings also showed that the content of Δ5-avenasterol was more significant in VAO than in VIO. Furthermore, cholesterol, Δ-7-stigmasterol, and Δ-7-avenasterol were the minor sterols found in all of the studied POs.

### 3.6. Tocopherols Content

Tocopherols, the main forms of vitamin E, are natural fat-soluble antioxidants that protect cells from the harmful effects of free radicals and other reactive oxygen species [27]. These components inhibit lipid oxidation in foods, thereby ensuring lipid stability during shelf life and determining the nutritional values of processed foods [64]. The results regarding the individual and total tocopherol content in the PO samples are given in Table 4.

Total tocopherol content (TTC) was found in a range of 49.29–719.36 mg/kg and 74.09–818.66 mg/kg in VIO and VAO, respectively. Thus, a high content of total tocopherol was observed in VAO compared to VIO. These levels were close to the reported values for argan oil (766 mg/kg) [67] and higher than those for olive oil (210.5 mg/kg) [68] and almond oil (371.13 mg/kg) [17]. These changes can be ascribed to certain climatic conditions, cultivar and species variations, and analytical circumstances. Thus, POs generated using the CP approach displayed the greatest TTC value, followed by those acquired using the Sox and Mac methods, respectively. In agreement with our present finding, Al Juhaimi et al. [11] reported that the TTC of eight tested nuts obtained using the Sox method was shown to be slightly lower than those from CP-nut oils.

Four types of tocopherols are present in PO, with α- and γ-tocopherols being the main components, representing about 91–95% of the total tocopherols, with the levels of γ- being higher in VIO than in α-tocopherol. On the other hand, α-tocopherol was present in much higher concentrations in VAO, followed by γ-tocopherol. Further, β- and δ-tocopherols were only detected at deficient levels, making up 4.1–7% of the tocopherol composition. These results agree with the findings of other authors [69,70]. Indeed, according to Casini et al. [71], tocopherol concentration rises with more precipitation and lower soil temperature. Although, Campos-Mondragón et al. [72] discovered that, based on the peanut cultivar type, α- and γ- tocopherols are reduced or increased during maturity.

As reflected in Table 4, significant variations were found within the contents of individual tocopherols among the different extraction methods. α- and γ- tocopherols were present at a significantly higher concentration in the VIO and VAO obtained via the CP method than from the equivalent using Sox and Mac methods, respectively. This finding is quite similar to the recent study of Al Juhaimi et al. [11], who demonstrated that the amounts of α- and γ- tocopherols in PO extracted via CP were 98.65 and 47.52 mg/kg, respectively. In contrast, that from the Sox-extracted oils was 82.49 and 42.61 mg/kg, respectively. Contrary to our study, Can-Cauich et al. [45] reported that the OS yielded pumpkin oil that was richer in α-tocopherol (28.39–37.45 mg/kg) compared to MP (12.96–23.68 mg/kg).

### 3.7. Correlation Analysis

Table 5 shows the Pearson correlation utilized to investigate the various factors examined in this work. The p-values of the correlation matrix coefficients among all variables were also computed and are presented in Table 6. A significant negative correlation was observed between the oleic (C18:1) and Linoleic (C18:2) (r^2^ = −0.999) fatty acids of the Pos extracted within the current study. The carotenoid content of the POs and oleic acid (C18:1) were also shown to be positively correlated (r^2^ = 0.892). The findings of the present work indicate that the correlation between PV and TOTOX was higher (r^2^ = 0.987) as compared to E_232_ of POs (r^2^ = 0.843).

Hence, the correlation analysis demonstrated that the p-AnV was positively related to E_270_ (r^2^ = 0.870). However, significant negative correlations were shown between the total tocopherols and AV (r^2^ = −0.975) and p-AnV (r^2^ = −0.952), respectively. Furthermore, the total tocopherols had a significant and direct correlation with the RSA (r^2^ = 0.953).

### 3.8. Principal Component Analysis (PCA)

**Figure 1:**
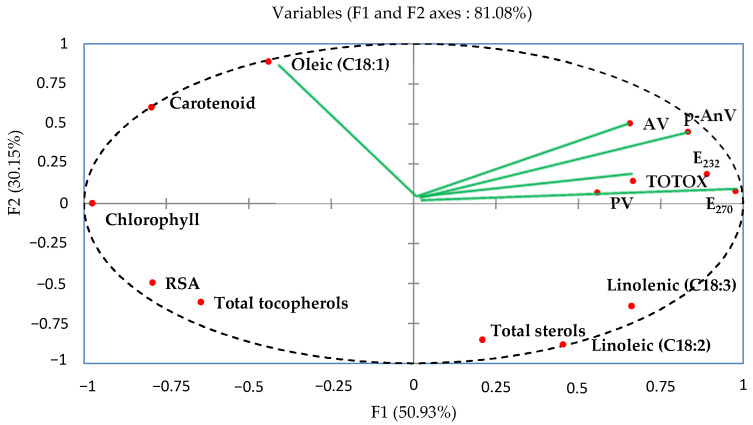
Principal component analysis (PCA) factorial plan.

The content of phytochemical compounds (pigment contents, PUFA, total sterols, and total tocopherols), analytical parameters (AV, PV, p-AnV, TOTOX, E_270_, and E_232_), and the radical scavenging activity (RSA) was submitted for principal component analysis (PCA). As seen in Figure 1, the PCA utilizes the F1 and F2 factorial plan to project the variables. The first principal component (F1) describes 50.93% of the variation, whilst the second main component (F2) represents the remaining 30.15%. The combined percentage of the two first primary components contributed to 81.08% of the total variation; therefore, its linear combination is reflective of the factors since it exceeds 50%. Further, the first two axes are adequate for representing the data in their entirety.

Figure 1 depicts the plane produced by the F1 and F2 axes, representing the variable correlation. The F1 axis is primarily formed by the positive association between AV, p-AnV, TOTOX, PV, E_232_, and E_270_, and the negative association between the total sterols, linoleic (C18:2), and linolenic (C18:3) fatty acids. Additionally, the variables RSA, total tocopherols, chlorophyll, and carotenoid contents, were scattered throughout the F2 axis on both the positive and negative flanks. These results are coherent with those of the correlation analysis.

**Figure 2:**
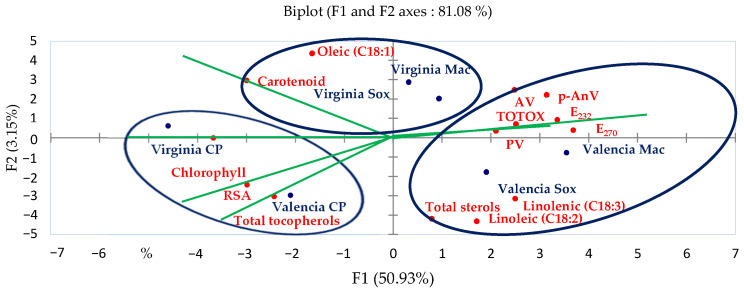
Individuals’ projection on the factorial plan (F1×F2). GI: Group I; GII: Group II; and GIII: Group III.

The biplot derived from the first two main components of the PCA is presented in Figure 2. Group “I” is made up of two individuals, “Virginia CP” and “Valencia CP”, which are distinguished by high levels of chlorophyll, total tocopherols, and RSA, as well as average levels of carotenoid and oleic acid. In addition, they present medium values of E_232_, E_270_, PV, p-AnV, and TOTOX.

Group “II” is formed by two samples, “Valencia Mac” and “Valencia Sox”. They are distinguished by a high amount of total sterol and PUFA. In addition, group “II” compared to group “I” showed a lower content of pigment, total tocopherols, and RSA.

Group “III” consists of two samples, “Virginia Sox” and “Virginia Mac”. Both samples’ carotenoid and oleic acid content are considerably greater than the others, but the levels of PUFA are lower than that of the different oil samples in group “II”. Additionally, they present average levels of E_232_ and E_270_ compared to group “II” and group “I”.

## 4. Conclusions

The present work investigated the effects of peanut varieties cultivated in Morocco and extraction techniques (CP, Sox, and Mac) on the yield, chemical profile, quality characteristics, and antioxidant activity of peanut seed oil. The FA composition showed slight differences between the extraction methods. Based on the findings of this study, it can be deduced that the CP method provides oils with better quality, higher amounts of bioactive compounds, and greater efficiency to scavenge DPPH radicals when compared to the other two processes. Further, the chemical composition of the fatty acids, phytosterols, and tocopherols varied among the two varieties. However, a greater level of oleic acid was obtained for the Virginia variety. Besides, a higher content of phytosterol and tocopherol was obtained from the Valencia variety. According to our study, the excellent shelf life afforded by CP peanut oil and other desired features makes the Moroccan PO a viable source of bioactive components with potential health benefits suitable for human nutrition and various industrial purposes.

## Figures and Tables

**Table 1:** Quality characteristics, pigments content, and radical scavenging activity of PO samples obtained by different extraction methods.

	Virginia			Valencia		
	CP	Sox	Mac	CP	Sox	Mac
AV (mg KOH/g)	0.50 ± 0.05 ^a^	0.59 ± 0.03 ^a^	0.81 ± 0.02 ^b^	0.46 ± 0.01 ^a^	0.56 ± 0.05 ^a^	0.80 ± 0.01 ^b^
PV (meq O_2_/kg)	2.15 ± 0.15 ^a^	12.9 ± 0.10 ^b^	4.25 ± 0.05 ^c^	3.7 ± 0.10 ^c^	11.8 ± 0.20 ^d^	5.25 ± 0.15 ^e^
p-AnV	0.84 ± 0.08 ^a^	3.20 ± 0.02 ^b^	3.77 ± 0.05 ^c^	0.75 ± 0.05 ^a^	2.13 ± 0.15 ^d^	4.68 ± 0.65 ^e^
TOTOX	5.14 ± 0.22 ^a^	29.00 ± 0.17 ^b^	12.27 ± 0.14 ^c^	8.15 ± 0.14 ^d^	25.73 ± 0.24 ^e^	15.18 ± 0.36 ^f^
E_232_	0.790 ± 0.002 ^a^	1.510 ± 0.001 ^b^	1.237 ± 0.001 ^c^	0.99 ± 0.005 ^d^	1.434 ± 0.005 ^e^	1.352 ± 0.004 ^f^
E_270_	0.034 ± 0.002 ^a^	0.131 ± 0.001 ^b^	0.104 ± 0.001 ^c^	0.051 ± 0.001 ^d^	0.132 ± 0.003 ^b^	0.183 ± 0.001 ^e^
Chlorophyll (mg/kg)	0.72 ± 0.00 ^a^	0.18 ± 0.02 ^b^	0.27 ± 0.01 ^c^	0.42 ± 0.01 ^d^	0.15 ± 0.00 ^b^	0.10 ± 0.02 ^b^
Carotenoid (mg/kg)	0.51 ± 0.00 ^a^	0.37 ± 0.02 ^bc^	0.4 ± 0.00 ^b^	0.29 ± 0.00 ^cd^	0.21 ± 0.02 ^de^	0.18 ± 0.02 ^e^
RSA (% inhibition DPPH)	47.68 ± 0.10 ^a^	28.58 ± 0.26 ^b^	11.17 ± 0.05 ^c^	50.10 ± 0.15 ^d^	30.17 ± 0.10 ^e^	12.66 ± 0.20 ^f^

Results are expressed as the mean values ± standard deviation of the three replicates (mean ± SD, n = 3); (a−f) different letters within a row indicate significant statistical differences (*p* ≤ 0.05). Acid value (AV); peroxide value (PV); p-anisidine value (p-AnV); totox value (TOTOX); conjugated dienes (E_232_); conjugated trienes (E_270_), and radical scavenging activity (RSA).

**Table 2:** Oil yield (%) and fatty acid composition (%) from seed oil extracted by various methods from two peanut varieties, Virginia and Valencia.

Fatty Acids (%)	Virginia			Valencia		
	CP	Sox	Mac	CP	Sox	Mac
Oil yield (%)	36.19 ± 0.89 ^a^	56.77 ± 1.37 ^b^	43.38 ± 1.14 ^c^	30.38 ± 0.16 ^d^	47.62 ± 0.24 ^c^	34.81 ± 1.04 ^ad^
Myristic (C14:0)	ND	0.02 ± 0.00 ^a^	0.03 ± 0.00 ^a^	ND	0.03 ± 0.00 ^a^	0.03 ± 0.00 ^a^
Palmitic (C16:0)	8.77 ± 0.25 ^a^	9.45 ± 0.01 ^b^	9.17 ± 0.01 ^c^	11.82 ± 0.02 ^d^	12.03 ± 0.00 ^e^	11.82 ± 0.02 ^d^
Palmitoleic (C16:1)	0.11 ± 0.01 ^a^	0.18 ± 0.00 ^b^	0.10 ± 0.00 ^ac^	0.08 ± 0.01 ^cd^	0.07 ± 0.00 ^de^	0.06 ± 0.01 ^e^
Margaric (C17:0)	0.06 ± 0.01 ^a^	0.03 ± 0.01 ^b^	0.10 ± 0.00 ^c^	0.05 ± 0.00 ^ab^	0.06 ± 0.00 ^a^	0.06 ± 0.00 ^a^
Heptadecenoid (C17:1)	ND	0.03 ± 0.00 ^b^	0.06 ± 0.01 ^c^	ND	0.02 ± 0.00 ^ab^	0.03 ± 0.01 ^b^
Stearic (C18:0)	4.38 ± 0.05 ^a^	3.20 ± 0.02 ^b^	3.50 ± 0.00 ^c^	3.35 ± 0.05 ^d^	3.36 ± 0.01 ^d^	3.59 ± 0.01 ^e^
Oleic (C18:1)	56.99 ± 0.01 ^a^	56.46 ± 0.01 ^b^	56.92 ± 0.03 ^a^	39.76 ± 0.02 ^c^	39.14 ± 0.01 ^d^	39.20 ± 0.01 ^d^
Linoleic (C18:2)	27.07 ± 0.01 ^a^	28.41 ± 0.01 ^b^	27.96 ± 0.01 ^c^	42.66 ± 0.01 ^d^	42.94 ± 0.01 ^e^	42.70 ± 0.01 ^d^
Linolenic (C18:3)	0.06 ± 0.00 ^a^	0.06 ± 0.00 ^a^	0.06 ± 0.00 ^a^	0.07 ± 0.00 ^a^	0.09 ± 0.00 ^b^	0.09 ± 0.00 ^b^
Arachidic C20:0	1.47 ± 0.00 ^a^	1.27 ± 0.00 ^b^	1.22 ± 0.01 ^c^	1.32 ± 0.01 ^d^	1.33 ± 0.01 ^d^	1.43 ± 0.01 ^e^
Eicosenoic (C20:1)	1.07 ± 0.01 ^a^	0.90 ± 0.00 ^b^	0.88 ± 0.00 ^c^	0.87 ± 0.00 ^c^	0.93 ± 0.00 ^d^	0.97 ± 0.00 ^e^
ƩSFA	14.69 ± 0.01 ^a^	13.95 ± 0.00 ^b^	13.99 ± 0.01 ^b^	16.55 ± 0.01 ^c^	16.79 ± 0.01 ^d^	16.9 ± 0.01 ^e^
ƩMUFA	58.18 ± 0.01 ^a^	57.54 ± 0.01 ^b^	57.90 ± 0.02 ^c^	40.71 ± 0.03 ^d^	40.13 ± 0.02 ^e^	40.24 ± 0.01 ^f^
ƩPUFA	27.13 ± 0.01 ^a^	28.46 ± 0.01 ^b^	28.02 ± 0.01 ^c^	42.73 ± 0.01 ^d^	43.02 ± 0.01 ^e^	42.79 ± 0.00 ^f^
ƩUFA	85.31 ± 0.02 ^a^	86.00 ± 0.01 ^b^	85.92 ± 0.01 ^c^	83.44 ± 0.01 ^d^	83.16 ± 0.01 ^e^	83.03 ± 0.01 ^f^
O/L ratio	2.11 ± 0.00 ^a^	1.99 ± 0.00 ^b^	2.03 ± 0.01 ^c^	0.93 ± 0.00 ^d^	0.91 ± 0.00 ^e^	0.92 ± 0.00 ^de^

Results are expressed as mean values ± standard deviation of three replicates (mean ± SD, n = 3); (a−f) different letters within a row indicate significant statistical differences (*p* ≤ 0.05). ND: Not detected.

**Table 3:** Phytosterol content (mg/kg) from oil extracted from the two peanut varieties, Virginia and Valencia, via different extraction methods.

Sterols (mg/kg)	Virginia			Valencia		
	CP	Sox	Mac	CP	Sox	Mac
Total sterols	1970.60 ± 4.2 ^a^	1904.16 ± 3.16 ^b^	1929.73 ± 3.96 ^c^	2816.34 ± 4.74 ^d^	2272.24 ± 3.74 ^e^	2676.88 ± 3.75 ^f^
Cholesterol	7.24 ± 0.00 ^a^	6.84 ± 0.01 ^b^	4.19 ± 0.01 ^c^	5.49 ± 0.01 ^d^	6.26 ± 0.00 ^e^	6.61 ± 0.01 ^f^
Campesterol	271.65 ± 0.36 ^a^	261.08 ± 0.26 ^b^	256.69 ± 0.3 ^c^	429.71 ± 0.35 ^d^	347.53 ± 0.34 ^e^	410.04 ± 0.36 ^f^
Stigmasterol	178.50 ± 0.25 ^a^	190.94 ± 0.08 ^b^	186.15 ± 0.14 ^c^	208.23 ± 0.21 ^d^	166.86 ± 0.19 ^e^	193.39 ± 0.14 ^f^
β-Sitosterol	1277.41 ± 1.81 ^a^	1205.25 ± 1.17 ^b^	1241.69 ± 2.01 ^c^	1839.42 ± 2.23 ^d^	1473.07 ± 1.94 ^e^	1740.68 ± 1.89 ^f^
Δ-5-avenosterol	170.08 ± 0.07 ^a^	169.63 ± 0.09 ^a^	163.53 ± 0.08 ^b^	250.62 ± 0.08 ^c^	208.29 ± 0.13 ^d^	236.62 ± 0.10 ^e^
Δ-7-stigmasterol	2.73 ± 0.01 ^a^	1.94 ± 0.01 ^b^	1.08 ± 0.01 ^c^	2.56 ± 0.02 ^d^	2.03 ± 0.03 ^b^	3.65 ± 0.01 ^e^
Δ-7-avenosterol	10.76 ± 0.01 ^a^	8.48 ± 0.01 ^b^	7.22 ± 0.01 ^c^	15.74 ± 0.06 ^d^	14.33 ± 0.01 ^e^	16.78 ± 0.02 ^f^

Results are expressed as mean values ± standard deviation of three replicates (mean ± SD, n = 3); (a−f) different letters within a row indicate significant statistical differences (*p* ≤ 0.05).

**Table 4:** Tocopherols content (mg/kg) in the oil extracted from two peanut varieties, Virginia and Valencia, via different extraction methods.

Tocopherol (mg/kg)	Virginia			Valencia		
	CP	Sox	Mac	CP	Sox	Mac
Total tocopherols	719.36 ± 2.39 ^a^	367.26 ± 1.96 ^b^	40.29 ± 2.02 ^c^	818.66 ± 1.79 ^d^	651.10 ± 2.08 ^e^	74.09 ± 1.56 ^f^
α-tocopherol	319.60 ± 1.04 ^a^	123.29 ± 0.62 ^b^	5.65 ± 0.36 ^c^	392.28 ± 0.73 ^d^	314.70 ± 0.81 ^e^	13.86 ± 0.52 ^f^
β-tocopherol	11.41 ± 0.11 ^a^	12.85 ± 0.09 ^b^	2.56 ± 0.16 ^b^	10.87 ± 0.17 ^a^	11.38 ± 0.07 ^a^	5.24 ± 0.15 ^c^
γ-tocopherol	343.68 ± 1.26 ^a^	214.62 ± 0.76 ^b^	30.91 ± 1.09 ^c^	371.71 ± 0.32 ^d^	309.03 ± 0.77 ^e^	54.84 ± 0.74 ^f^
δ-tocopherol	26.16 ± 0.34 ^a^	12.21 ± 0.07 ^b^	0.00 ± 0.00 ^c^	29.78 ± 0.13 ^d^	15.71 ± 0.16 ^e^	0.00 ± 0.00 ^c^

Results are expressed as mean values ± standard deviation of three replicates (mean ± SD, n = 3); (a−f) different letters within a row indicate significant statistical differences (*p* ≤ 0.05).

**Table 5:** The coefficient of Pearson’s correlation matrix between the variables: quality parameters (AV, PV, p-AnV, TOTOX, E_232_, and E_270_), chlorophyll and carotenoid contents, total sterols, total tocopherols, and unsaturated fatty acids: oleic (C18:1), Linoleic (C18:2), and Linolenic (C18:3) of the different PO samples.

Variables	Total Sterols	Total Tocopherols	Oleic (C18:1)	Linoleic (C18:2)	Linolenic (C18:3)	AV	PV	p-AnV	TOTOX	E_232_	E_270_	Chlorophyll	Carotenoid	RSA
Total sterols	**1**													
Total tocopherols	0.204	**1**												
Oleic (C18:1)	**−0.885**	−0.215	**1**											
Linoleic (C18:2)	**0.884**	0.205	−**0.999**	**1**										
Linolenic (C18:3)	0.621	−0.049	−**0.879**	**0.871**	**1**									
AV	−0.106	−**0.975**	0.088	−0.082	0.181	**1**								
PV	−0.253	−0.036	−0.083	0.102	0.244	−0.057	**1**							
p-AnV	−0.076	−**0.952**	0.006	0.004	0.278	**0.926**	0.252	**1**						
TOTOX	−0.252	−0.193	−0.077	0.096	0.277	0.101	**0.987**	0.406	**1**					
E_232_	−0.102	−0.515	−0.177	0.200	0.393	0.453	**0.843**	0.705	**0.914**	**1**				
E_270_	0.138	−0.680	−0.351	0.360	0.626	0.671	0.549	**0.870**	0.664	**0.869**	**1**			
Chlorophyll	−0.188	0.577	0.415	−0.437	−0.573	−0.567	−0.652	−0.767	−0.744	−**0.939**	−**0.924**	**1**		
Carotenoid	−0.705	0.162	**0.892**	−**0.900**	−**0.903**	−0.245	−0.362	−0.401	−0.408	−0.580	−0.719	0.778	**1**	
RSA	0.167	**0.953**	−0.045	0.034	−0.261	−**0.963**	−0.206	−**0.961**	−0.356	−0.664	−0.794	0.730	0.348	**1**

The values in bold are different from 0 at a significance level α = 0.05.

**Table 6:** p-values of all variable coefficients in the correlation matrix.

Variables	Total Sterols	Total Tocopherols	Oleic (C18:1)	Linoleic (C18:2)	Linolenic (C18:3)	AV	PV	p-AnV	TOTOX	E_232_	E_270_	Chlorophyll	Carotenoid	RSA
Total sterols	**0**													
Total tocopherols	0.698	**0**												
Oleic (C18:1)	**0.019**	0.683	**0**											
Linoleic (C18:2)	**0.019**	0.697	**<0.001**	**0**										
Linolenic (C18:3)	0.188	0.926	**0.021**	**0.024**	**0**									
AV	0.841	**0.001**	0.869	0.878	0.732	**0**								
PV	0.629	0.947	0.875	0.848	0.641	0.915	**0**							
p-AnV	0.885	**0.003**	0.991	0.994	0.593	**0.008**	0.630	**0**						
TOTOX	0.629	0.714	0.885	0.856	0.595	0.848	**0.000**	0.425	**0**					
E_232_	0.848	0.296	0.737	0.704	0.440	0.367	**0.035**	0.118	**0.011**	**0**				
E_270_	0.794	0.137	0.495	0.483	0.184	0.145	0.259	**0.024**	0.150	**0.025**	**0**			
Chlorophyll	0.721	0.230	0.413	0.386	0.235	0.241	0.161	0.075	0.090	**0.006**	**0.009**	**0**		
Carotenoid	0.118	0.759	**0.017**	**0.014**	**0.014**	0.640	0.481	0.431	0.422	0.227	0.107	0.069	**0**	
RSA	0.751	**0.003**	0.933	0.948	0.618	**0.002**	0.695	**0.002**	0.489	0.151	0.059	0.100	0.499	**0**

The values in bold are different from 0 at a significance level α = 0.05.

## Data Availability

All data generated or analyzed during this study are included in this published article.

## Abbreviations

CP	Cold press
Sox	Soxhlet
Mac	Maceration
VIO	Virginia oil
VAO	Valencia oil
PO	Peanut oil
FA	Fatty acid
FAME	Fatty acid methyl ester
MUFA	Monounsaturated fatty acids
SFA	Saturated fatty acids
PUFA	Polyunsaturated fatty acids
PS	Phytosterols
TTC	Total tocopherol content
DPPH	1,1-diphenylDiphenyl-2-picrylhydrazylPicrylhydrazyl
AV	Acid value
PV	Peroxide value
p-AnV	*p*-Anisidine value
PCA	Principal component analysis
HPLC	High-performance liquid chromatography
O/L	Oleic/Linoleic acid
